# Sentiment analysis of internet posts on vaccination using ChatGPT and comparison with actual vaccination rates in South Korea

**DOI:** 10.12688/f1000research.145845.2

**Published:** 2024-09-02

**Authors:** Sunyoung Park

**Affiliations:** 1 Department of Psychiatry, National Health Insurance Service Ilsan Hospital, Goyang-si, Gyeonggi-do, 10444, South Korea

**Keywords:** COVID-19, vaccination, ChatGPT, sentiment analysis

## Abstract

**Background:**

This study used ChatGPT for sentiment analysis to investigate the possible links between online sentiments and COVID-19 vaccination rates. It also examines Internet posts to understand the attitudes and reasons associated with vaccine-related opinions.

**Methods:**

We collected 500,558 posts over 60 weeks from the Blind platform, mainly used by working individuals, and 854 relevant posts were analyzed. After excluding duplicates and irrelevant content, attitudes toward and reasons for vaccine opinions were studied through sentiment analysis. The study further correlated these categorized attitudes with the actual vaccination data.

**Results:**

The proportions of posts expressing positive, negative, and neutral attitudes toward COVID-19 vaccines were 5%, 83%, and 12%, respectively. The total post count showed a positive correlation with the vaccination rate, indicating a high correlation between the number of negative posts about the vaccine and the vaccination rate. Negative attitudes were predominantly associated with societal distrust and perceived oppression.

**Conclusions:**

This study demonstrates the interplay between public perceptions of COVID-19 vaccines as expressed through social media and vaccination behavior. These correlations can serve as useful clues for devising effective vaccination strategies.

## Introduction

The exchange of opinions and information on social media platforms has become vital for social interaction and communication. This transformation has created an environment in which information and opinions about societal issues spread rapidly and are shared. ^1^ In recent years, the global landscape has been significantly affected by the COVID-19 pandemic, leading to an abundance of Internet posts discussing various aspects of this crisis. Among these critical topics, vaccination has emerged as an indispensable tool for overcoming the challenges posed by the pandemic. ^2^ Despite their importance, the distribution and vaccination rates vary significantly across nations, regions, and occupations, ^3^ and these differences are closely related to individuals’ perceptions of and attitudes toward vaccines. ^4^^,^^5^

Gathering public opinion on vaccination through Internet posts is anticipated to contribute to providing accurate information and obtaining insights for policy formulation in infectious disease prevention. ^6^ Sentiment analysis is commonly employed to analyze extensive and diverse online content. It is a form of text classification that focuses on subjective statements and is often called opinion mining. The goal was to analyze opinions to gain insights into public perceptions. ^7^ However, the complexity and necessity of meticulous processes to enhance accuracy pose challenges for sentiment analysis. ^8^ Moreover, the language used on social media platforms can vary in style among users and may require understanding within the context. In such cases, existing sentiment-analysis models may find it challenging to adapt to diversity and changes. ^9^

Therefore, in this study, we employed ChatGPT, a state-of-the-art language model recognized for its natural language understanding capabilities, to explore the nuanced insights derived from the analysis. ^10^ ChatGPT excels in contextual understanding, allowing comprehension of the nuanced meanings within the context of a conversation. This contextual awareness is particularly valuable in sentiment analysis, where the interpretation of sentiments often relies on an understanding of the surrounding text. In addition, ChatGPT is adaptable to diverse language nuances, capturing the intricacies of expression across different linguistic styles, cultural variations, and demographic factors. ^11^ This adaptability can be crucial in sentiment analysis, especially when dealing with social media discourse, where language can be highly dynamic and varied.

In particular, the sentiment analysis conducted in this study using ChatGPT holds several strengths. Firstly, the objective was to compare the attitudes towards vaccines identified in online content with actual vaccination rates. While analyzing data extracted from internet posts through sentiment analysis contributes to understanding a multitude of opinions, there has been a limitation in verifying how these expressed opinions relate to real-world behavioral outcomes. Analyzing the relationship with societal behavioral outcomes, as reflected in actual vaccination rates, will aid in comprehending the results of sentiment analysis and considering future analytical directions. ^12^ Secondly, sentiment analysis conducted in Korean, a relatively smaller cultural sphere, has encountered limitations despite various approaches. This study sought to explore the extent to which ChatGPT could contribute to understanding the subtle nuances in internet posts written by diverse individuals in the Korean language. ^13^

This study aimed to conduct a sentiment analysis of Internet postings using OpenAI GPT-3.5-turbo model, exploring the potential associations between diverse opinions expressed on social media and actual vaccination rates. The goal was to investigate the causal relationship between sentiments observed through sentiment analysis and real-world behavior. We hypothesized that a higher prevalence of positive vaccine-related posts correlates with elevated vaccination rates. Additionally, the research intended to further analyze the reasons associated with attitudes toward vaccines as expressed in internet posts.

## Literature review

Recent studies have provided significant insights by analyzing public sentiment towards COVID-19 vaccines on social media. These studies primarily identify positive or negative reactions to evaluate public perceptions of the vaccine. ^14^ Sentiment analysis of tweets using natural language processing techniques has been effective in analyzing changing attitudes toward vaccines over time and across different countries. Analyzing 11 million tweets from 180 countries revealed an increasing trend in positive responses to vaccines over time. ^15^ Furthermore, it was found that providing sufficient information is essential for fostering positive attitudes toward vaccines. ^16^

Such data is crucial for formulating public health policies and communication strategies related to vaccines. When examining the correlation between these results and actual vaccination rates, it was reported that higher numbers of positive tweets correlate with higher vaccination rates. ^15^ Additionally, studies have shown that regions with more positive tweets tend to have higher vaccination rates, especially among populations aged 40 and above. ^17^ However, distrust and misinformation also contribute to lower vaccination rates. ^18^ Therefore, providing accurate information and fostering positive public sentiment are essential to increase vaccination rates.

To achieve reliable results in these studies, effective sentiment analysis is essential. However, sentiment analysis of tweets presents several challenges, such as sarcasm, irony, and language-specific challenges. ^19^ Current natural language processing technologies like Vader and TextBlob are mostly optimized for English data, making them difficult to use for analyzing minor languages like Korean. ^20^ Even when new Korean lexicons are developed for research, their accuracy ranges from 40-70%, which leads to inconsistent results. ^21^ Therefore, advanced AI-based natural language processing tools like ChatGPT, which excel in understanding complex language patterns and nuances, show promise in achieving high accuracy across various languages and dialects. ^22^ This capability is expected to enhance the reliability of sentiment analysis.

## Methods

This observational study analyzed users’ perceptions of the COVID-19 vaccine on Internet platforms targeting working individuals and their relationship with actual vaccination rates. This study, involving the collection and analysis of publicly available internet posts without containing personal information, received approval for a consent waiver and exemption from review through the National Health Insurance Service Ilsan Hospital Institutional Review Board (IRB) (NHIMC-2023-08-028).

### Web crawling and data collection

On the Internet, there are cases in which individuals intentionally post multiple messages to emphasize their claims or engage in actions with specific intentions, such as marketing. ^23^ To counteract this form of online manipulation, we utilized web crawling on the social network service (SNS) ‘Blind ( https://www.teamblind.com/kr/),’ where individuals involved in employment, job-seeking, and workplace organizations actively participate and interact. Users join this SNS by using their individual email accounts associated with their respective workplaces, anonymously. The posts were web-scraped using the Python Selenium package, adhering to the Blind’s Access Restriction Protocol (robots. txt). The data collection period spanned between March 23, 2022, and May 16, 2023, totaling 60 weeks, and a total of 500,558 posts were gathered. Information such as post number, posting date, publicly available workplace information of the post author, post title, and content was collected through web scraping. To ensure the integrity of the dataset, we implemented a robust method for excluding duplicate posts. Each post was uniquely identified based on a combination of attributes, including post number, posting date, and author details. Posts sharing identical attributes were flagged as duplicates, and only the earliest occurrence was retained for analysis. This process aimed to eliminate redundancy and maintain the diversity of opinions within the dataset. After then, posts were filtered based on the presence of keywords associated with COVID-19, such as “COVID,” “coronavirus,” and vaccine-specific terms.scraping. To ensure the integrity of the dataset, we implemented a robust method for excluding duplicate posts. Each post was uniquely identified based on a combination of attributes, including post number, posting date, and author details. After then, posts were filtered on whether they contained the following keywords. Keywords related to COVID-19 were checked, such as COVID-19, COVID, and coronavirus, as well as keywords related to vaccination, for example, vaccination, vaccine, and inoculation. Python’s Beautiful Soup libarary and Request module were used for this purpose. Keyword searches were conducted on the title and content of each post.

### Data refinement and pre-processing

After excluding duplicate posts and those less relevant to COVID-19, 4,419 posts were curated, with 854 posts specifically mentioning the vaccines chosen for the analysis. The posts underwent text preprocessing, involving UTF-8 encoding, stop word and URL removal, as well as the removal of emojis and special characters.

### Sentiment analysis and reasoning extraction using ChatGPT

Subsequently, the posts were analyzed using the OpenAI GPT-3.5-turbo model. This model, pretrained on a large corpus of language data by OpenAI, classified the attitude expressed in each post toward the vaccine as positive, negative, or neutral, following the criteria commonly used in the sentiment analysis of conditional statements, evaluating whether the sentiment toward a specific topic is positive, negative, or neutral. ^7^ In cases where the model could not confidently assign an attitude, we instructed it to respond with ‘unclear’.

Using ChatGPT, we extracted the reasons for positive or negative attitudes toward vaccines from Internet posts. The system’s role was an AI assistant tasked with identifying the reasons behind the positive or negative views on COVID-19 vaccines in the given posts. The goal was to extract up to three reasons per post. Cases in which the reasons were unknown or difficult to classify were noted accordingly.

### Statistical analysis

COVID-19 vaccination information was collected from the Korea Disease Control and Prevention Agency daily vaccination status ( https://ncv.kdca.go.kr/vaccineStatus.es?mid=a11710000000). The counts for the first and second doses were collected over the 60-week research period, including additional winter booster vaccination counts for 31 weeks starting on October 11, 2022.

Considering the working patterns of employees and the lower frequency of vaccination on weekends, a correlation analysis was performed on the sum of the posts and weekly vaccination counts. Furthermore, an association analysis was conducted to explore relationships among the reasons mentioned in the posts, specifically focusing on understanding the relationships between key reasons behind positive and negative attitudes. All analyses were performed using R version 4.2.2.

## Results

The analysis used OpenAI’s gpt-3.5-turbo model, which yielded 851 emotional evaluations. In three cases, the model reported uncertainty; upon manual review, these instances were deemed uncertain and excluded from the analysis. Two psychiatrists evaluated 100 posts, resulting in 85% agreement with the results provided by the gpt-3.5-turbo model. The results of the confusion matrix are presented in the Extended data.

Over the 60-week analysis period, the sentiment distribution was as follows: 44 positive (5%), 704 negative (83%), and 103 neutral (12%) posts. For posts collected over 31 weeks starting from October 11, 2022, and associated with additional vaccinations during the winter season, the sentiment distribution was 20 positive (6%), 254 negative (80%), and 43 neutral (14%) posts. The total vaccination counts, weekly vaccination averages, and weekly post-vaccination averages for each study period are presented in Table 1.

**Table 1.** The mean of posts by sentiment and vaccination types.

A. Whole study period (for 60 weeks)							
	Posts				Vaccination		
	Positive (N=44)	Negative (N=704)	Neutral (N=103)	Total (N=851)	1 ^st^ Dose (N=179053)	2 ^nd^ Dose (N=224735)	Total (N=403788)
Mean ± SD (per week)	0.73 ± 1.01	11.73 ± 8.31	1.72 ± 1.74	14.18 ± 9.67	2984.22 ± 4520.73	3745.58 ± 4038.60	6729.80 ± 8053.79

B. Winter booster vaccination period (for 31 weeks)					
	Postings				Winter booster vaccination (N=6682925)
	Positive (N=20)	Negative (N=254)	Neutral (N=43)	Total (N=317)	
Mean ± SD (per week)	0.65 ± 0.97	8.19 ± 6.28	1.39 ± 1.45	10.23 ± 7.32	228841.41 ± 205373.47

SD, Standard Deviation.

### Relationship between attitudes toward COVID-19 vaccination in internet postings and actual vaccination rates

**Figure 1.**
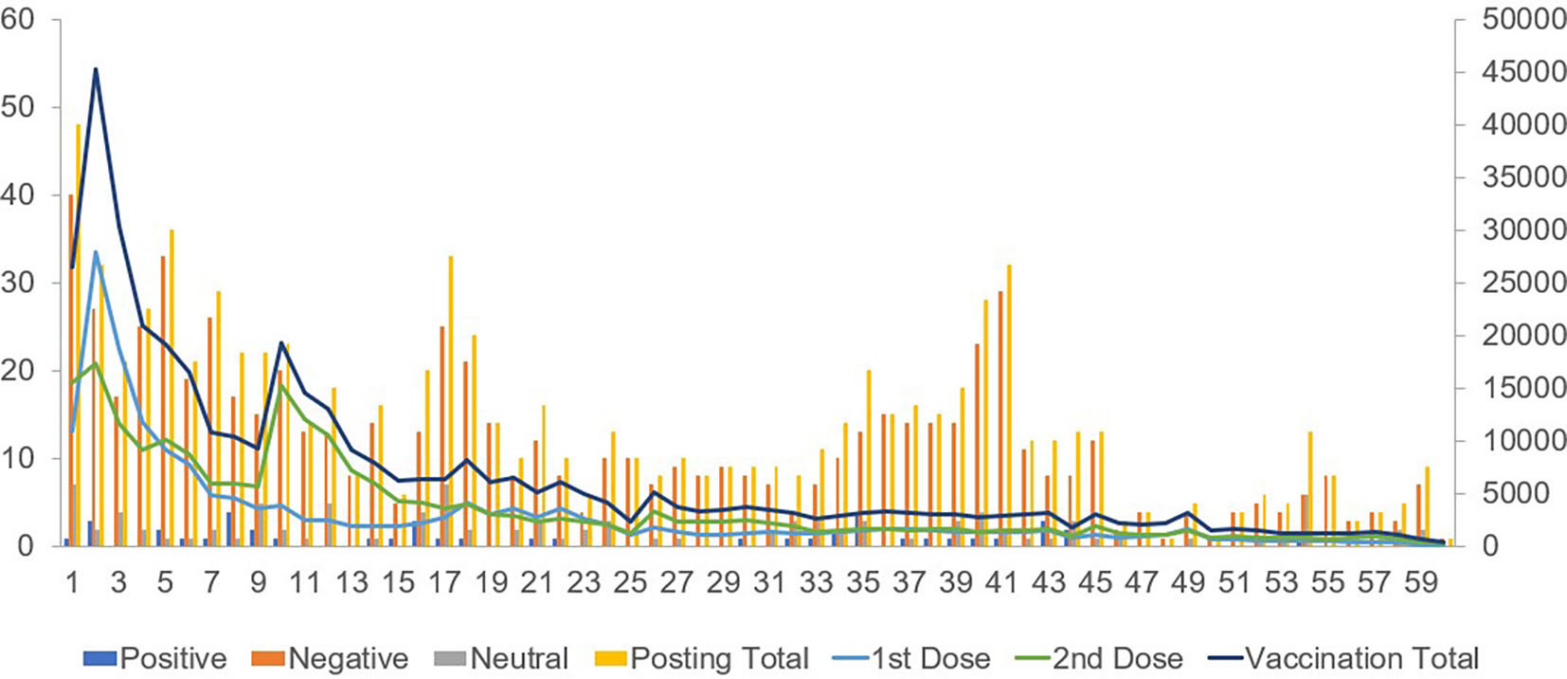
Comparison between the number of posts about vaccination (left axis) and actual vaccination (right axis) for 60 weeks.

Positive posts regarding vaccination attitudes showed a weak positive correlation with the first-dose vaccination counts. Negative posts on vaccines exhibited a moderately positive correlation with both first- and second-dose vaccinations. Posts expressing a neutral attitude showed a weak positive correlation with second-dose vaccinations ( Table 2, Figure 1).

**Table 2.** Correlation between number of vaccinations and postings by attitude toward vaccines.

	1 ^st^ Dose	2 ^nd^ Dose	Total	Booster vaccination
Positive attitude	0.27 ^*^	0.22	0.26 ^*^	0.50 ^**^
Negative attitude	0.59 ^**^	0.66 ^**^	0.66 ^**^	0.69 ^**^
Neutral attitude	0.24	0.31 ^*^	0.29 ^*^	0.18
Total number of postings	0.58 ^**^	0.65 ^**^	0.65 ^**^	0.69 ^**^

** The correlation is significant at the 0.01 level (2-tailed).

* The correlation is significant at the 0.05 level (2-tailed).

Regarding the booster vaccination count during the winter season, a strong correlation was observed between positive and negative posts regarding vaccination attitudes.

The total counts of positive, negative, and neutral posts showed strong correlations with the counts of the first, second, and booster vaccinations. Table 2 presents the correlation results.

### Extraction of attitudinal reasons toward vaccination and association analysis between reasons

Using ChatGPT, we extracted the reasons for positive or negative attitudes toward vaccines from Internet posts. Among the 44 positive posts, the most prevalent (73%) was prevention, cited in 39 instances. For the 704 negative posts, the most common reason (28%) was distrust of the social system, which was mentioned in 194 cases. Two psychiatrists manually reviewed 100 posts and compared the outcomes in the extracted results. For the first reason per post, there were 58 instances of agreement, 37 for the second reason, and 14 for the third reason.

An association analysis was also conducted for the reasons mentioned in the postings. However, this aspect was excluded from further study because of the low agreement rate (14%) for the third reason per post during the manual review. Cases in which reasons showed a high frequency of repetition were classified into item categories. The positive reasons for vaccination include prevention, symptom alleviation, reduced mortality rate, effectiveness, safety, fewer side effects, containment of the spread of infection, and immune system reinforcement. Negative reasons for vaccination included six items: mistrust of the social system, antipathy toward social oppression, side effects, concerns about side effects, lack of information, and perception of insufficient efficacy. If the first and second reasons for a post fell under the aforementioned classification, they were included in the association analysis. In the study of positive posts, 36 groups were used to analyze positive posts, and 381 groups were analyzed.

In the analysis of positive reasons, “Decreasing mortality rate” is associated with “Symptom alleviation,” showcasing a support of 5.71%, confidence of 50.00%, and a lift of 2.92. Additionally, instances of “Post-recovery symptom alleviation” (support = 5.71%, confidence = 100.00%, lift = 1.21%), “Immune activity strengthening” (support = 17.14%, confidence = 100.00%, lift = 1.21%), “Inhibition of infection spread” (support = 31.43%, confidence = 100.00%, lift = 1.21%), and “Safety” (support = 14.29%, confidence = 83.33%, lift = 1.01%) are correlated with the item of “Prevention.”

**Figure 2.**
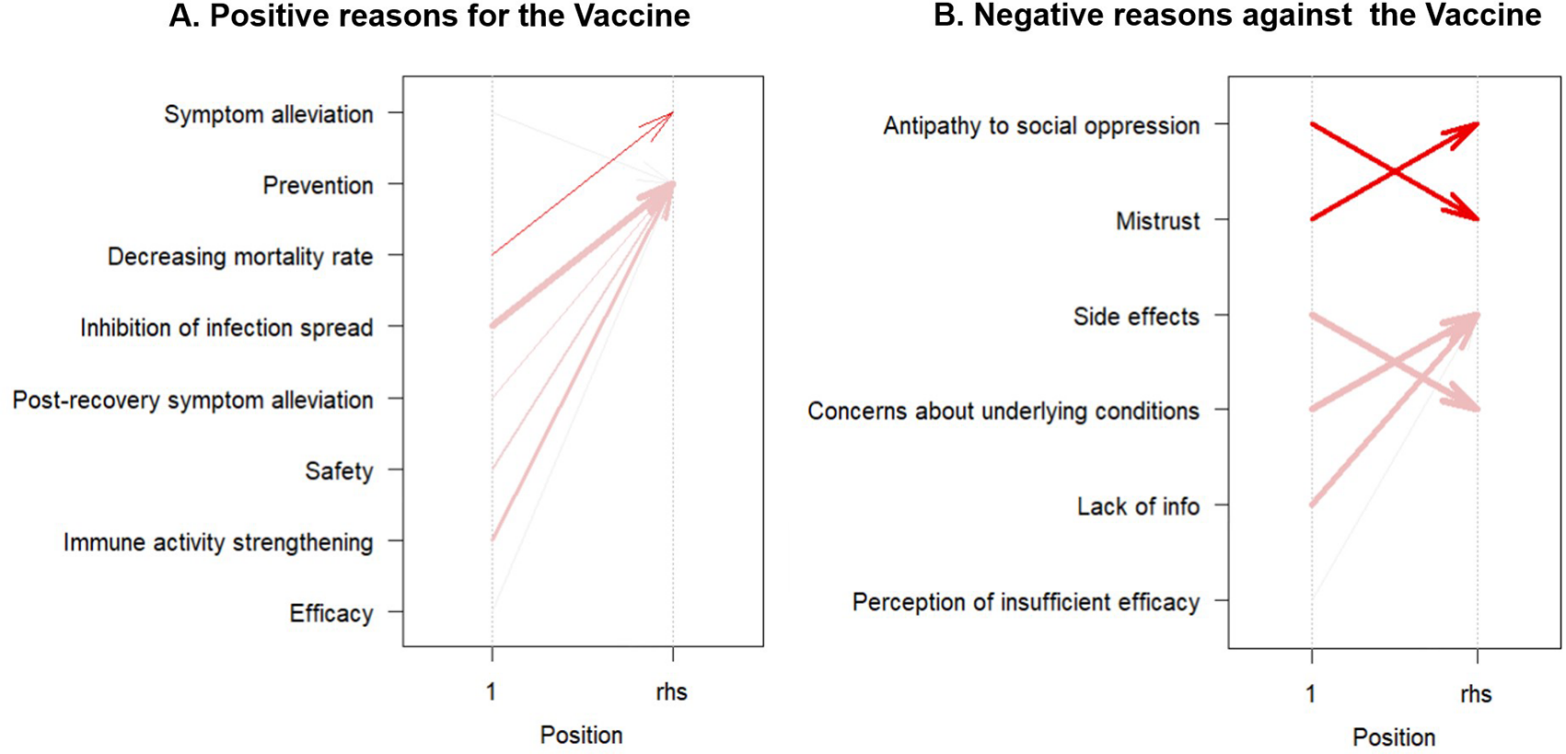
Association rules visualization for vaccine sentiments: positive (A) and negative (B) reasons.

In the analysis of negative reasons, ‘Antipathy to social oppression’ strongly correlates with ‘Mistrust’ (support = 21.78%, confidence = 94.32%, lift = 2.16). Conversely, ‘Mistrust’ is moderately associated with ‘Antipathy to social oppression’ (support = 21.78%, confidence = 50.00%, lift = 2.16). Instances involving concerns about underlying conditions were linked to side effects (support = 29.13%, confidence = 91.74%, lift = 1.26). Conversely, side effects were associated with concerns about the underlying conditions (support = 29.13%, confidence = 40.07%, lift = 1.26). The presence of “lack of information” was strongly associated with”unknown side effects” (support = 23.62%, confidence = 90.91%, lift = 1.25). The overall trends in the association analyses are shown in Figure 2.

## Discussion

This study explored the relationship between professionals’ attitudes toward the COVID-19 vaccine as expressed in internet posts and actual vaccination rates. Additionally, it examined the positive and negative reasons associated with vaccine attitudes and investigated the relationships between them.

The sentiment analysis conducted using OpenAI’s gpt-3.5-turbo model demonstrated an 85% concordance rate when compared with evaluations by mental health specialists. This result indicates a substantial level of accuracy of the model and affirms the utility of automated analysis using natural language processing technology. Recent studies using deep-learning-based analysis models have reported sentiment analysis accuracies ranging from approximately 70% to 90%. These studies often evaluate sentiments in posts related to COVID-19 on major social media platforms, such as Twitter. ^24^^,^^25^ In particular, the accuracy for positive posts was significantly decreased in this study. Data imbalances in sentiment analysis could be a cause of this, leading to models overfitting. This results in proficient detection of negative sentiments—due to their prevalence in training datasets—and reduced accuracy in identifying positive sentiments. ^26^ The reasoning analysis in this study, which aimed at extracting the reasons behind positive or negative sentiments toward vaccines, showed a lower matching rate (58%). Compared to sentiment analysis, which gauges the overall sentiment, reasoning analysis delves into specific words or phrases that explain why a sentiment is expressed. This process involves a deeper understanding of language nuances and contextual cues, making it a more intricate task. ^27^ Furthermore, ChatGPT tends to include irrelevant content when producing results for short posts, making it challenging to infer the reasons. This behavior is likely attributable to the generative nature of ChatGPT, a characteristic inherent in creative AI models. ^11^

Previous survey studies have shown that negative opinions towards vaccines accounted for 39.8%, ^28^ whereas in the present study, negative opinions are markedly higher at 82%. These results are associated with certain social phenomena in which negative or extreme online content is prevalent. Cyber venting ^29^^,^^30^ is a phenomenon in which internet users express dissatisfaction, stress, or anger online, making it easy to express various negative emotions related to vaccination, discomfort about side effects, and social pressure. Moreover, the freedom to swiftly respond and exchange opinions through comments in anonymous online spaces can result in uninhibited emotional expressions. ^31^^,^^32^ This phenomenon was evident in the posts analyzed in this study, in which various expressions of anxiety and discomfort regarding vaccine side effects, derogatory remarks about compliant attitudes toward vaccination, and rumors related to vaccines were observed.

In this study, the correlation analysis between the number of posts and vaccine doses administered indicated a strong correlation between the total number of positive, negative, and neutral posts and the counts of the first, second, and additional vaccine doses. Numerous studies have explored the relevance of social media platforms to epidemiological patterns and medical information. ^33^ For instance, even before the COVID-19 pandemic, research suggested a strong correlation between the regional distribution of social media posts related to infectious diseases and their actual spread. ^34^ Additionally, studies on trend analysis using search queries have shown associations with the spread of infectious diseases. ^35^ In this study, the diverse opinions expressed online before and after the vaccination could be considered a reflection of the various perspectives emerging on online platforms. The strong correlation between these negative opinions and actual vaccination rates can be inferred from the content of internet posts. In Korea, the total vaccination rate reported by the Korea Disease Control and Prevention Agency was very high at 96.9%. However, the content of internet posts revealed instances where individuals were vaccinated due to social discomfort or job requirements, as well as due to social pressure. Additionally, posts expressing social discomfort due to refusal to vaccinate were also found.

In this study, implications were derived by analyzing the underlying factors influencing attitudes toward vaccines. For positive attitudes toward vaccines, individuals expected preventive effects, personal relief from symptoms and after-effects, and societal benefits, such as preventing the spread of infection. In contrast, negative attitudes toward vaccines were associated with resentment and distrust toward social oppression. This aligns with discussions in numerous studies during the pandemic, indicating that public distrust of efforts to prevent epidemics at the societal level can lead to strong resistance. ^36^^–^^38^ Information gaps in various media channels regarding vaccines can reinforce public anxiety and contribute to negative attitudes. ^39^ Therefore, it is crucial to implement effective communication strategies and educational initiatives to address these concerns and promote a more informed perspective on vaccination. ^40^^,^^41^

The limitations of this study include its focus on SNS users among employed individuals, warranting future research that encompasses diverse demographic groups and regions for a more comprehensive understanding. Furthermore, research incorporating various factors is essential to investigate the relationship between opinion formation on Internet platforms and vaccination behavior. Additionally, limited number of posts poses significant constraints on this study, compared to other studies. ^6^^,^^34^ This limitation may be attributed to the fact that the BLIND community, which was the focus of the analysis, primarily targets professionals, resulting in a predominance of posts related to salary, career, and other job-related topics. The period of the study, which occurred about one year after the initial vaccine rollout in February 2022, coincided with a significant decrease in public interest in vaccines, which also likely contributed to the fewer posts related to vaccines.

Moreover, the analysis of sentiment analysis models, including the gpt-3.5-turbo model, reveals additional limitations. These models may not fully capture the subtle nature of emotions, and the interpretation of emotional expressions can vary among individuals. ^11^ Therefore, involving a diverse range of reviewers across different age groups and backgrounds for manual review is crucial. Considering the gpt-3.5-turbo model, there is a limitation in fully understanding subtle nuances and cultural contexts. This is particularly evident in contexts like Korea, characterized by unique language styles, cultural norms, and demographic characteristics. ^13^

In conclusion, this study revealed the interaction between the public’s perception of the COVID-19 vaccine expressed on social media and their actual vaccination behavior. The perception of risk and willingness to be vaccinated can be influenced by various mass media sources, such as the news. Opinions encountered on SNS, which people use, are also likely to significantly impact individuals’ perceptions of vaccines due to biased approaches to information and the phenomenon of conformity. Therefore, implementing social strategies that provide appropriate vaccine information in an accessible manner is crucial.

### Ethical considerations

This study was exempted from review by the National Health Insurance Ilsan Hospital Institutional Review Board (NHIMC-2023-08-028) 04/09/2023.

## Data Availability

Zenodo: Korea Disease Control and Prevention Agency daily COVID19 vaccination status, https://zenodo.org/doi/10.5281/zenodo.10252895. ^42^ Vaccination data is accessible in the form of an Excel file. This file comprehensively includes vaccination rate information relevant to the research findings. COVID-19 vaccination information was collected from the Korea Disease Control and Prevention Agency daily vaccination status ( https://ncv.kdca.go.kr/vaccineStatus.es?mid=a11710000000). For additional details or specific requests regarding data provision, feel free to contact us. The data used for SNS crawling in this study, acquired through social media crawling, cannot be shared due to ethical and copyright restrictions related to social media content. A comprehensive description of the methodology is presented in the Methods section, along with the Python code below, facilitating the replication of the study. For inquiries concerning the methodology, please direct any questions to the corresponding author. https://github.com/bechungan/Scraping-and-Sentiment-Analysis-using-CGPT . Zenodo: STROBE checklist for Sentiment analysis of internet posts on vaccination using ChatGPT and comparison with actual vaccination rates in South Korea, https://doi.org/10.5281/zenodo.10429910. Zenodo: The result of the confusion matrix for the accuracy evaluation of 100 posts. This project contains the data: confusion matrix.docx. https://doi.org/10.5281/zenodo.13381133. ^43^

## References

[1] Choi S : The roles of media capabilities of smartphone-based SNS in developing social capital. *Behav. Inform. Technol.* 2019;38(6):609–620. 10.1080/0144929X.2018.1546903

[2] Razai MS Chaudhry UAR Doerholt K : Covid-19 vaccination hesitancy. *BMJ.* 2021;373. 10.1136/bmj.n1138 34016653

[3] Noushad M Rastam S Nassani MZ : A global survey of COVID-19 vaccine acceptance among healthcare workers. *Front. Public Health.* 2021;9:794673. 35211453 10.3389/fpubh.2021.794673 PMC8860987

[4] Adane M Ademas A Kloos H : Knowledge, attitudes, and perceptions of COVID-19 vaccine and refusal to receive COVID-19 vaccine among healthcare workers in northeastern Ethiopia. *BMC Public Health.* 2022;22(1):128. 10.1186/s12889-021-12362-8 35042476 PMC8765812

[5] Jing R Fang H Wang H : The role of general attitudes and perceptions towards vaccination on the newly-developed vaccine: Results from a survey on COVID-19 vaccine acceptance in China. *Front. Psychol.* 2022;13:841189. 10.3389/fpsyg.2022.841189 35712143 PMC9194573

[6] Griffith J Marani H Monkman H : COVID-19 vaccine hesitancy in Canada: Content analysis of tweets using the theoretical domains framework. *J. Med. Internet Res.* 2021;23(4):e26874. 10.2196/26874 33769946 PMC8045776

[7] Taboada M : Sentiment Analysis: An Overview from Linguistics. *Annu. Rev. Linguist.* 2016;2:325–347. 10.1146/annurev-linguistics-011415-040518

[8] Kenyon-Dean K Ahmed E Fujimoto S , editors. Sentiment analysis: It’s complicated! *Proceedings of the 2018 Conference of the North American Chapter of the Association for Computational Linguistics: Human Language Technologies, Volume 1 (Long Papers).* 2018.

[9] Birjali M Kasri M Beni-Hssane A : A comprehensive survey on sentiment analysis: Approaches, challenges and trends. *Knowl. Based Syst.* 2021;226:107134. 10.1016/j.knosys.2021.107134

[10] Orrù G Piarulli A Conversano C : Human-like problem-solving abilities in large language models using ChatGPT. *Front. Artif. Intell.* 2023;6:1199350. 10.3389/frai.2023.1199350 37293238 PMC10244637

[11] Kalla D Smith N : Study and Analysis of Chat GPT and its Impact on Different Fields of Study. *Int. J. Innov. Sci. Res. Technol.* 2023;8(3). 10.1007/978-3-031-43803-5

[12] Wankhade M Rao ACS Kulkarni C : A survey on sentiment analysis methods, applications, and challenges. *Artif. Intell. Rev.* 2022;55(7):5731–5780. 10.1007/s10462-022-10144-1

[13] Lee G-m Song S : Can Korean Language Models Detect Social Registers in Utterances? *Korean J. Appl. Linguist.* 2023;48(2):585–605.

[14] Çılgın C Gökçen H Gökşen Y : Sentiment analysis of public sensitivity to COVID-19 vaccines on Twitter by majority voting classifier-based machine learning Twitter’da COVID-19 aşılarına karşı kamu duyarlılığının çoğunluk oylama sınıflandırıcısı temelli makine öğrenmesi ile duygu analizi. *J. Fac. Eng. Archit. Gazi Univ.* 2023;38(2):1093–1104. 10.17341/gazimmfd.1030198

[15] Wang H Li Y Hutch MR : Patterns of diverse and changing sentiments towards COVID-19 vaccines: a sentiment analysis study integrating 11 million tweets and surveillance data across over 180 countries. *J. Am. Med. Inform. Assoc.* 2023;30(5):923–931. 10.1093/jamia/ocad029 36821435 PMC10114113

[16] Greyling T Rossouw S : Positive attitudes towards COVID-19 vaccines: A cross-country analysis. *PLoS One.* 2022;17(3): e0264994. 10.1371/journal.pone.0264994 35271637 PMC8912241

[17] Cheng T Han B Liu Y : Exploring public sentiment and vaccination uptake of COVID-19 vaccines in England: a spatiotemporal and sociodemographic analysis of Twitter data. Front. Public Health. 2023;11:1193750. 10.3389/fpubh.2023.1193750 37663835 PMC10470640

[18] Osuji VC Galante EM Mischoulon D : COVID-19 vaccine: A 2021 analysis of perceptions on vaccine safety and promise in a US sample. *PLoS One.* 2022;17(5): e0268784. 10.1371/journal.pone.0268784 35587947 PMC9119541

[19] Wankhade M Rao ACS Kulkarni C : A survey on sentiment analysis methods, applications, and challenges. *Artif. Intell. Rev.* 2022;55(7):5731–5780. 10.1007/s10462-022-10144-1

[20] Kim Y Kang M Jeong SR : Text mining and sentiment analysis for predicting box office success. *KSII T Internet Info.* 2018;12(8):4090–4102.

[21] Jang H Shin H : Effective use of linguistic features for sentiment analysis of korean. Proceedings of the 24th Pacific Asia Conference on Language, Information and Computation.Waseda University.2011.

[22] Sudirjo F Diantoro K Al-Gasawneh JA : Application of ChatGPT in Improving Customer Sentiment Analysis for Businesses. *JTEKSIS.* 2023;5(3):283–288. 10.47233/jteksis.v5i3.871

[23] Lee S : Detection of political manipulation in online communities through measures of effort and collaboration. *ACM Trans. Web.* 2015;9(3):1–24. 10.1145/2767134

[24] Chakraborty K Bhatia S Bhattacharyya S : Sentiment Analysis of COVID-19 tweets by Deep Learning Classifiers—A study to show how popularity is affecting accuracy in social media. 2020;97:106754. 10.1016/j.asoc.2020.106754 PMC7521435 33013254

[25] Liu C Fang F Lin X : Improving sentiment analysis accuracy with emoji embedding. *J. Saf. Sci. Resil.* 2021;2(4):246–252. 10.1016/j.jnlssr.2021.10.003

[26] Kochanek M Cichecki I Kaszyca O : Improving Training Dataset Balance with ChatGPT Prompt Engineering. *Electronics.* 2024;13(12):2255. 10.3390/electronics13122255

[27] Zhou F Jianxin Jiao R Linsey JS : Latent customer needs elicitation by use case analogical reasoning from sentiment analysis of online product reviews. *J. Mech. Des.* 2015;137(7):071401. 10.1115/1.4030159

[28] Hwang SE Kim W-H Heo J : Socio-demographic, psychological, and experiential predictors of COVID-19 vaccine hesitancy in South Korea, October-December 2020. *Hum. Vaccin. Immunother.* 2022;18(1):1–8. 10.1080/21645515.2021.1983389 34614382 PMC8920123

[29] Rösner L Krämer NC : Verbal venting in the social web: Effects of anonymity and group norms on aggressive language use in online comments. *Soc. Media Soc.* 2016;2(3):205630511666422. 10.1177/2056305116664220

[30] Rodríguez-Hidalgo C Tan ES Verlegh PW : Expressing emotions in blogs: The role of textual paralinguistic cues in online venting and social sharing posts. *Comput. Hum. Behav.* 2017;73:638–649. 10.1016/j.chb.2017.04.007

[31] Suler J : The online disinhibition effect. *Cyberpsychol. Behav.* 2004;7(3):321–326. 10.1089/1094931041291295 15257832

[32] Lapidot-Lefler N Barak A : Effects of anonymity, invisibility, and lack of eye-contact on toxic online disinhibition. *Comput. Hum. Behav.* 2012;28(2):434–443. 10.1016/j.chb.2011.10.014

[33] Mkhize PL : Effect of social trust on health information exchange in social network sites. *S. Afr. J. Inf. Manag.* 2023;25(1):1539. 10.4102/sajim.v25i1.1539

[34] Signorini A Segre AM Polgreen PM : The Use of Twitter to Track Levels of Disease Activity and Public Concern in the U.S. during the Influenza A H1N1 Pandemic. *PLoS One.* 2011;6(5):e19467. 10.1371/journal.pone.0019467 21573238 PMC3087759

[35] Mavragani A Gkillas K : COVID-19 predictability in the United States using Google Trends time series. *Sci. Rep.* 2020;10(1):20693. 10.1038/s41598-020-77275-9 33244028 PMC7692493

[36] Lin C Parker T Pejavara K : “I Would Never Push a Vaccine on You”: A Qualitative Study of Social Norms and Pressure in Vaccine Behavior in the US. *Vaccines.* 2022;10(9):1402. 10.3390/vaccines10091402 36146480 PMC9502292

[37] Wu Z-X Zhang H-F : Peer pressure is a double-edged sword in vaccination dynamics. *EPL.* 2013;104(1):10002. 10.1209/0295-5075/104/10002

[38] Decoteau CL Sweet PL : Vaccine Hesitancy and the Accumulation of Distrust. *Soc. Probl.* 2023;spad006. 10.1093/socpro/spad006

[39] Lee W Ahn S : Risk Perception and Vaccination Intention towards COVID-19 News. *Korean Journal of Journalism & Communication Studies.* 2022;66(6):388–425. 10.20879/kjjcs.2022.66.6.011

[40] Palmedo PC Rauh L Lathan HS : Exploring distrust in the wait and see: Lessons for vaccine communication. *Am. Behav. Sci.* 2022;000276422110628. 10.1177/00027642211062865

[41] Larson HJ Jarrett C Eckersberger E : Understanding vaccine hesitancy around vaccines and vaccination from a global perspective: a systematic review of published literature, 2007–2012. *Vaccine.* 2014;32(19):2150–2159. 10.1016/j.vaccine.2014.01.081 24598724

[42] Korea Disease Control and Prevention Agency: Korea Disease Control and Prevention Agency daily COVID19 vaccination status (https://ncv.kdca.go.kr/vaccineStatus.es?mid=a11710000000). Zenodo.[Dataset].2023. 10.5281/zenodo.10252896

[43] Sunyoung P : The result of the confusion matrix for the accuracy evaluation of 100 posts. Zenodo. 2024. 10.5281/zenodo.13381133

