# Supplementary material for: Sentiment analysis of internet posts on vaccination using ChatGPT and comparison with actual vaccination rates in South Korea
Source: F1000Res. 2025 Sep 24;13:96. Originally published 2024 Feb 15. [Version 4] doi: 10.12688/f1000research.145845.4 (PMC12179586; doi:10.12688/f1000research.145845.4)
Supplement: Peer review history

# Reviewer report: Reviewer response for version 4

**DOI:** 10.5256/f1000research.186240.r417210

**Published:** 2025-10-14

**Authors:** Ummu Fatihah Mohd Bahrin

**Affiliations:** 1 Universiti Teknologi MARA Cawangan Terengganu, Kuala Terengganu, Malaysia

I have carefully gone through the revisions made, and I am satisfied with the improvements. The authors have sufficiently addressed my previous comments, and I have no further reservations.

Is the work clearly and accurately presented and does it cite the current literature?

Yes

If applicable, is the statistical analysis and its interpretation appropriate?

I cannot comment. A qualified statistician is required.

Are all the source data underlying the results available to ensure full reproducibility?

Partly

Is the study design appropriate and is the work technically sound?

Yes

Are the conclusions drawn adequately supported by the results?

Yes

Are sufficient details of methods and analysis provided to allow replication by others?

Yes

Reviewer Expertise:

NA

I confirm that I have read this submission and believe that I have an appropriate level of expertise to confirm that it is of an acceptable scientific standard.

# Reviewer report: Reviewer response for version 3

**DOI:** 10.5256/f1000research.176895.r382353

**Published:** 2025-06-19

**Authors:** Young Anna Argyris

**Affiliations:** 1 Michigan State University, East Lansing, Michigan, USA

**Overview**

 This paper addresses a crucial issue—public sentiment toward COVID-19 vaccination in South Korea—and examines it through a novel approach: utilizing ChatGPT-based sentiment analysis on social media data. The study's attempt to connect sentiment trends to real-world vaccination rates is timely and commendable, and the use of advanced language models is a noteworthy methodological innovation.

 However, the manuscript has several substantive issues that prevent it from being indexed in its current form. While I highly appreciate the authors’ effort and believe this line of inquiry is valuable, the concerns outlined below—including methodological limitations and unclear contributions —need to be addressed more fully. I share these critiques in the hope that they will help the authors improve their paper.

**Literature Review**

 The authors may also consider referencing a similar approach in Argyris et al.'s study published in Social Science & Medicine, which also used natural language processing to analyze online vaccine discourse: (Ref.1). They also demonstrate significant correlations between social media posts and vaccination rates, and elaborate potential reasons for the associations, which might be helpful for the authors to contextualize the contribution of this paper within the broader literature. 

**Methods**

*Note: Other reviewers have already addressed other issues, such as data imbalance and small sample size, and are not reiterated here.*

 The authors specify that posts were collected from Blind, an anonymous social media community targeted at working professionals. While this is helpful, the implications of using a niche platform with a specific user demographic should be discussed more thoroughly, particularly regarding the representativeness of the data, ethical considerations related to consent and privacy, and the generalizability of the study’s findings. To their credit, the authors do acknowledge this limitation in the discussion section, noting that the Blind platform primarily targets professionals and may not capture broader public sentiment. However, merely acknowledging this limitation does not fully address the methodological implications. The restricted demographic may systematically bias the types of sentiments expressed, especially given that workplace pressures and professional norms likely influence how vaccination is discussed on the platform. Blind is organized into numerous company- and industry-specific groups, which may affect the topics and tone of discussions, as well as amplify particular workplace-related sentiments such as coercion or pressure to comply with vaccination mandates.

 The posts underwent text preprocessing, which involved UTF-8 encoding, removal of stop words and URLs, as well as removal of emojis and special characters. While such cleaning is common, the removal of emojis may be questionable, as emojis—though not crucial—can be useful for sentiment analysis and emotional nuance detection. The authors should justify this decision or acknowledge its potential implications for the analysis.

 A major concern is the reliability of ChatGPT in accurately classifying sentiment in the analyzed posts. The authors report an 85% agreement rate between the model and two psychiatrists, but this evaluation was conducted on only 100 posts out of 854 analyzed. Given the complexity and nuance of language in social media discourse—particularly when dealing with sarcasm, irony, or emotionally charged content—a validation sample of 100 posts is insufficient to establish confidence in the model's overall performance. A more rigorous evaluation, ideally with interrater comparisons across a larger and more diverse subset of posts, is necessary to support the claim that the model accurately reflects user sentiment.

 The authors should clarify the coding scheme.

**Results and Visualization**

 Figure 2 is very difficult to understand. Based on the accompanying description, the authors appear to be using association rule mining to identify co-occurring sentiment categories within the reasons given for or against vaccination. For example, they report support, confidence, and lift values to indicate the strength and reliability of relationships like “Decreasing mortality rate” being associated with “Symptom alleviation,” or “Antipathy to social oppression” with “Mistrust.” However, the figure itself lacks clear labels, an intuitive layout, and an adequate explanation in the caption. The connection between the visual elements and these statistical measures is not readily apparent. The figure would benefit from a simplified design, a clearer legend, and a more detailed narrative in the main text to help readers accurately interpret the findings. For guidance on more effective visualizations of association rule mining results, the authors may refer to Dolores et al. (2023) - (Ref 2). Table 4, which presents a clear and practical summary of association rule metrics and their interpretations.

**Discussion**

 Interestingly, the study finds a strong correlation between the number of negative posts and actual vaccination rates, despite the overwhelmingly negative sentiment expressed in the data. The authors acknowledge that many individuals were vaccinated not out of conviction, but because of job requirements or social pressure. In this context, the findings suggest that high vaccination rates in Korea (reported at 96.9%) may reflect compliance driven by external pressures rather than positive public sentiment. This nuance is important: public health behavior may not align with expressed beliefs, particularly in environments where vaccination is perceived as socially or professionally obligatory. The authors could emphasize this tension more clearly to avoid the mistaken interpretation that high uptake equates to high trust or positive attitudes toward vaccines.

Is the work clearly and accurately presented and does it cite the current literature?

No

If applicable, is the statistical analysis and its interpretation appropriate?

Partly

Are all the source data underlying the results available to ensure full reproducibility?

Partly

Is the study design appropriate and is the work technically sound?

No

Are the conclusions drawn adequately supported by the results?

No

Are sufficient details of methods and analysis provided to allow replication by others?

No

Reviewer Expertise:

Information Systems, Health Communication, Computational method

I confirm that I have read this submission and believe that I have an appropriate level of expertise to confirm that it is of an acceptable scientific standard, however I have significant reservations, as outlined above.

## References

[1] : Vaccine rhetoric on social media and COVID-19 vaccine uptake rates: A triangulation using self-reported vaccine acceptance. *Social Science & Medicine* .2024;348: 10.1016/j.socscimed.2024.116775 10.1016/j.socscimed.2024.116775 PMC1107782438579627

[2] : A big data association rule mining based approach for energy building behaviour analysis in an IoT environment. *Scientific Reports* .2023;13(1) : 10.1038/s41598-023-47056-1 10.1038/s41598-023-47056-1 37957251 PMC10643377

# Response

**Published:** 2025-08-06

**Authors:** Sunyoung Park

**Affiliations:** Psychiatry, National Health Insurance Service Ilsan Hospital, Goyang-si, Gyeonggi-do, South Korea

1. Literature Review: Reference to Argyris et al. : Thank you for the suggestion. We agree that the study by Argyris et al. provides important contextual support for our work. We have revised the Literature Review section to include and discuss this reference. Specifically, we highlight how our study contributes to the growing body of research connecting social media sentiment with real-world vaccine behaviors, with a distinct focus on Korean-language data and the use of ChatGPT.

 2. Methods – Use of Blind and Demographic Limitations: This is an important point. While we acknowledged this limitation, we agree that the methodological implications should be expanded. We have added a detailed discussion in both the Methods and Discussion sections, emphasizing how Blind’s focus on professionals may introduce bias, particularly regarding expressions of workplace-related pressure to vaccinate. We also discuss how company- and industry-specific groupings may affect discourse tone and topic.

 3. Removal of Emojis in Preprocessing: We thank the reviewer for this valuable observation. We have now acknowledged in the Methods section that while emojis can contribute emotional context to sentiment analysis, they were removed to reduce preprocessing complexity and ensure consistency in textual inputs for the language model. We have also included a note in the Limitations section about the possible loss of emotional signals due to this decision.

 4. Validation Sample Size and Coding Scheme: Thank you for this insightful comment. In response, we have expanded the validation sample from 100 to 200 posts to improve the reliability of our sentiment classification. The additional validation yielded a concordance rate of 84% between the two board-certified psychiatrists, which is consistent with the initial agreement rate.

 We have also provided a more detailed description of our coding scheme in the Methods section. Posts were categorized as positive, negative, or neutral based on the emotional tone and content related to COVID-19 vaccination. Annotation was guided by clinical criteria commonly used in psychiatric evaluation of affective expression, including linguistic cues (e.g., appraisal, sarcasm, urgency), expressed intentions (e.g., compliance, avoidance), and context (e.g., workplace influence, medical risk). Disagreements between annotators were resolved through discussion and consensus. These revisions are now reflected in the updated manuscript.

 5. Figure 2 – Difficult Interpretation of Association Rules: Thank you for pointing this out. We have redesigned Figure 2 using a more intuitive layout and clearer legends. The caption has been expanded to explain what each element represents (support, confidence, lift), and we’ve added a narrative in the Results section that walks through key associations.

 6. Discussion – Clarify the Tension Between Negative Sentiment and High Vaccination Rates: We fully agree. In the revised Discussion, we now highlight this discrepancy more explicitly. We discuss how high vaccine uptake in Korea may have been driven by workplace mandates or social expectations rather than positive sentiment, reinforcing the complex dynamics between public behavior and online discourse.

 7. Reproducibility and Methodological Transparency: To enhance reproducibility, we have updated our GitHub repository to include the full set of preprocessing scripts, sentiment analysis prompts used with ChatGPT, and R code used for association rule mining. We have described the process of categorizing variables for reasoning analysis in more detail in the main text. We have also clarified in the manuscript that the SNS post data cannot be shared due to ethical and copyright concerns. However, we ensured that sufficient methodological details are provided to allow replication of our process using alternative datasets.

# Reviewer report: Reviewer response for version 2

**DOI:** 10.5256/f1000research.169571.r336916

**Published:** 2024-11-25

**Authors:** Ummu Fatihah Mohd Bahrin

**Affiliations:** 1 Universiti Teknologi MARA Cawangan Terengganu, Kuala Terengganu, Malaysia

The study notes that the sentiment analysis faced challenges due to the data imbalance, which led to overfitting, in detecting negative sentiments while reducing accuracy for positive feelings. However, the document does not elaborate on specific strategies to address the imbalance. Recommendations for Handling Imbalanced Datasets

 Resampling Techniques:

 1)Oversampling: Increase the number of positive and neutral posts using techniques like SMOTE (Synthetic Minority Oversampling Technique).

 2)Undersampling: Reduce the number of negative posts to balance the dataset while ensuring that critical information is retained.

Is the work clearly and accurately presented and does it cite the current literature?

Yes

If applicable, is the statistical analysis and its interpretation appropriate?

I cannot comment. A qualified statistician is required.

Are all the source data underlying the results available to ensure full reproducibility?

Partly

Is the study design appropriate and is the work technically sound?

Yes

Are the conclusions drawn adequately supported by the results?

Yes

Are sufficient details of methods and analysis provided to allow replication by others?

Yes

Reviewer Expertise:

Setimnet Mining, machine learning, deep learning.

I confirm that I have read this submission and believe that I have an appropriate level of expertise to confirm that it is of an acceptable scientific standard, however I have significant reservations, as outlined above.

# Response

**Published:** 2025-01-09

**Authors:** Sunyoung Park

**Affiliations:** Psychiatry, National Health Insurance Service Ilsan Hospital, Goyang-si, Gyeonggi-do, South Korea

Thank you so much for taking the time to review my manuscript and for providing such valuable and thoughtful comments. Your insights have been incredibly helpful and offered me a great opportunity to deepen my understanding of the topic. I truly appreciate the effort you put into making this a better piece of research.

 Following your comment, I considered several ways to fix the data imbalance and conducted additional analyses. I have also added the results and acknowledged this as a limitation in the Discussion section.

**1. Limitations of Oversampling and Undersampling**

1. **Undersampling**: Since the total amount of data in this study is not sufficient, reducing the number of negative postings would lead to a lack of data for correlation analysis, which could undermine the reliability of the results. Therefore, undersampling was deemed unsuitable for this study.
2. **Oversampling**: The recommended method, such as SMOTE, produces results in vector form, making it difficult to apply to sentiment analysis using language models like ChatGPT. For this reason, we attempted data augmentation using ChatGPT ^1^.

**2. Data Augmentation**

- Data augmentation using ChatGPT was conducted with controlled prompts to encourage the use of synonyms and increase the diversity of positive and neutral data while maintaining contextual consistency.
- The augmented data was adjusted so that positive and neutral data accounted for 25-30% of the total dataset, with the following distribution after augmentation:
  - Positive: Increased from 44 to 264 (220 added)
  - Neutral: Increased from 103 to 295 (192 added)
  - Negative: Maintained at 704
- The augmented data was randomly mixed with the original data, forming the full dataset, and sentiment analysis was conducted again using ChatGPT.

**3. Results of Sentiment Analysis After Data Augmentation**

- **Results**: After data augmentation, the accuracy of the sentiment analysis did not improve. On the contrary, actual neutral data was more frequently misclassified as negative.
- **Analysis of Causes**: We propose the following hypotheses to explain these results:
  1. **Nondeterministic Nature of ChatGPT**: ChatGPT generates nondeterministic responses ^2^, which may have led to the augmented minor dataset having no significant impact on the analysis results. This nondeterministic characteristic is particularly prone to inaccuracies in borderline cases (where the sentiment is ambiguous between positive and negative) and sarcasm.
  2. **Quality Degradation in Augmented Data**: The initial sentiment analysis accuracy was approximately 84%, suggesting possible inaccuracies in borderline cases and sarcastic expressions. If data augmentation was based on these inaccurate results, it likely generated lower-quality data that further reduced the overall dataset accuracy. The increase in cases where neutral data was misclassified as negative indicates potential challenges in accurately identifying ambiguous boundaries.

**References**

 1.         Dai H, Liu Z, Liao W, Huang X, Cao Y, Wu Z, et al. Auggpt: Leveraging chatgpt for text data augmentation. *arXiv. *2023; preprint arXiv:230213007. 10.48550/arXiv.2302.13007

 2.         Ouyang T, MaungMaung A, Konishi K, Seo Y, Echizen I. Stability analysis of chatgpt-based sentiment analysis in ai quality assurance. *Electronics*. 2024;13(24):5043. 10.3390/electronics13245043

# Reviewer report: Reviewer response for version 2

**DOI:** 10.5256/f1000research.169571.r319806

**Published:** 2024-09-04

**Authors:** Cihan Cilgin

**Affiliations:** 1 Bolu Abant Izzet Baysal University, Bolu, Turkey

I think it is appropriate to approve and index this study in its current, final form.

Is the work clearly and accurately presented and does it cite the current literature?

No

If applicable, is the statistical analysis and its interpretation appropriate?

Yes

Are all the source data underlying the results available to ensure full reproducibility?

Partly

Is the study design appropriate and is the work technically sound?

Partly

Are the conclusions drawn adequately supported by the results?

Yes

Are sufficient details of methods and analysis provided to allow replication by others?

Partly

Reviewer Expertise:

Machine Learning, Deep Learning, sentiment analysis,

I confirm that I have read this submission and believe that I have an appropriate level of expertise to confirm that it is of an acceptable scientific standard.

# Reviewer report: Reviewer response for version 1

**DOI:** 10.5256/f1000research.159851.r284069

**Published:** 2024-06-17

**Authors:** Cihan Cilgin

**Affiliations:** 1 Bolu Abant Izzet Baysal University, Bolu, Turkey

This study used ChatGPT to perform sentiment analysis on posts about Covid-19 vaccines.

 1. Within the scope of the study, many studies conducted within the same scope were ignored and the literature section was not given. However, the existing studies in the literature are very important to both reveal and support the current contributions of these studies. For example, the study given below is a direct alternative to this issue:

 >> Çılgın, C., Gökçen, H., & Gökşen, Y. (2023 [Ref -1]). Sentiment analysis of public sensitivity to COVID-19 vaccines on Twitter by majority voting classifier-based machine learning. *Journal of the Faculty of Engineering and Architecture of Gazi University*, *38*(2)

 2. There are many repetitive sentences under the title of web crawling and data collection. This title should definitely be reviewed again.

 3.  It is really interesting that only 854 relevant posts were confiscated out of approximately 500 thousand posts. Many studies in the literature used social media posts much higher than this number. The reader should be made more aware here by giving more details about this filtering process.

 4. Using a lexicon-based approach such as Vader or TextBlob in sentiment analysis, in addition to ChatGPT, may make the results of this study more valuable. In addition, a suitable data set is available for such a method comparison.

 5. Presenting a confusion matrix of the results obtained as a result of the 100 posts considered for the test data set is very important in terms of the consistency of the results.

 6. There are repetitive expressions, especially in the Discussion section of the study.

 7. There is no statement in the Discussion section that compares the findings of this study with the findings of other studies in the literature. As mentioned before, this is related to the lack of literature section. The similarity or difference of the findings with the existing literature is very important for the reader to evaluate the findings of this study. This study used ChatGPT to perform sentiment analysis on posts about Covid-19 vaccines.

Is the work clearly and accurately presented and does it cite the current literature?

No

If applicable, is the statistical analysis and its interpretation appropriate?

Yes

Are all the source data underlying the results available to ensure full reproducibility?

Partly

Is the study design appropriate and is the work technically sound?

Partly

Are the conclusions drawn adequately supported by the results?

Yes

Are sufficient details of methods and analysis provided to allow replication by others?

Partly

Reviewer Expertise:

Machine Learning, Deep Learning, sentiment analysis,

I confirm that I have read this submission and believe that I have an appropriate level of expertise to confirm that it is of an acceptable scientific standard, however I have significant reservations, as outlined above.

## References

[1] : Twitter’da COVID-19 aşılarına karşı kamu duyarlılığının çoğunluk oylama sınıflandırıcısı temelli makine öğrenmesi ile duygu analizi. *Gazi Üniversitesi Mühendislik Mimarlık Fakültesi Dergisi* .2022;38(2) : 10.17341/gazimmfd.1030198 1093-1104 10.17341/gazimmfd.1030198

# Response

**Published:** 2024-08-04

**Authors:** Sunyoung Park

**Affiliations:** Psychiatry, National Health Insurance Service Ilsan Hospital, Goyang-si, Gyeonggi-do, South Korea

Thank you for your comment. I have revised this article as below.

 Please go through the respective Author responses for Reviewer Comments made.

 1. As suggested, I have developed and included a comprehensive literature review related to the main topic of this study. Additionally, I have incorporated important insights from the paper you mentioned as an example, which have significantly contributed to the argument presented in my work.

 2. Thank you for pointing this out. There was an error in the editing process of the Methods section, which led to the repetition of content. This has now been corrected.

 3. Thank you for highlighting this critical point. The relatively small number of relevant posts collected in this study can be attributed to the characteristics of the website BLIND, from which the data was gathered, and the fact that data collection occurred some time after vaccines were a hot topic. I have added a detailed explanation to address this. Additionally, I have also enhanced and elaborated on the filtering process in the study.

 4. Thank you for your insightful suggestion. It is true that most sentiment analysis studies utilize English data, and the lexicons you mentioned are indeed based on English. However, Korean, being a less commonly used language globally, presents challenges in applying these pre-established analytic methods directly. I believe this underscores the significance of using ChatGPT in our study, which is specifically adapted to handle Korean text. This point has been detailed in the literature review and discussed further in the discussion section of our paper.

 5. I have added the results of the confusion matrix and included comments on the identified features in the discussion section. Additionally, while creating the confusion matrix, there were slight modifications to the previously manually evaluated accuracy results (from 86% to 85%).

 6. I have revised the Discussion section to eliminate repetitive expressions and streamline the content.

 7. Thank you for your meaningful feedback. Our study has notable differences from other studies, particularly due to the linguistic and sociocultural factors specific to Korea. I have addressed these differences and added a comparison of our findings with those in the existing literature in the Discussion section.
